# On-chip particle levitation and micromanipulation using bulk acoustic waves

**DOI:** 10.1039/d5lc00747j

**Published:** 2025-10-02

**Authors:** Emilie Vuille-dit-Bille, Marc-Alexandre Dubois, Junsun Hwang, Dara Bayat, Thomas Overstolz, Amit Dolev, Sarah Heub, Gilles Weder, Michel Despont, Mahmut Selman Sakar

**Affiliations:** a CSEM SA Neuchâtel Switzerland thomas.overstolz@csem.ch; b Institute of Mechanical Engineering, EPFL Lausanne Switzerland selman.sakar@epfl.ch

## Abstract

Acoustofluidic technologies enable precise manipulation of microscale objects using travelling and standing sound waves in physiological fluids, offering exciting capabilities for biomedical and chemical applications. In particular, surface acoustic wave-based devices have shown great promise for on-chip micromanipulation, but their planar transducer configuration limits the usable workspace near the microchannel surface. Here, we present a novel acoustofluidic platform based on a digitally addressable array of piezoelectric micromachined ultrasound transducers (PMUTs) that generate bulk acoustic waves and acoustic traps within three-dimensional (3D) fluidic chambers. Through a combination of finite element modelling and experimental measurements, we quantify the acoustic field distribution and study acoustic trap formation dynamics. We demonstrate deterministic 3D levitation of particles in water at rest and under continuous flow by generating standing acoustic waves across the height of the chamber. Our results show that 30 μm polystyrene particles can be levitated to a pressure node generated 640 μm above the surface with less than 6% positional error. The system applies in-plane acoustic radiation forces as high as 90 pN to keep the particles in the trap under flow rates up to 40 μL min^−1^. We leverage spatiotemporal modulation of the acoustic field for continuous planar transport of microparticle aggregates. PMUT arrays are microfabricated using conventional cleanroom techniques, thus can be readily integrated with compact fluidic systems. Our work lays the foundation for the development of reconfigurable and scalable acoustofluidic micromanipulation systems, with broad potential for lab-on-chip technologies.

## Abstract 2

We introduce an acoustic micromanipulation platform that precisely levitates and transports particles in water within a 3D microfluidic chamber. We demonstrate its potential as a phased array system for complex manipulation tasks at rest and in-flow.

## Graphical abstract

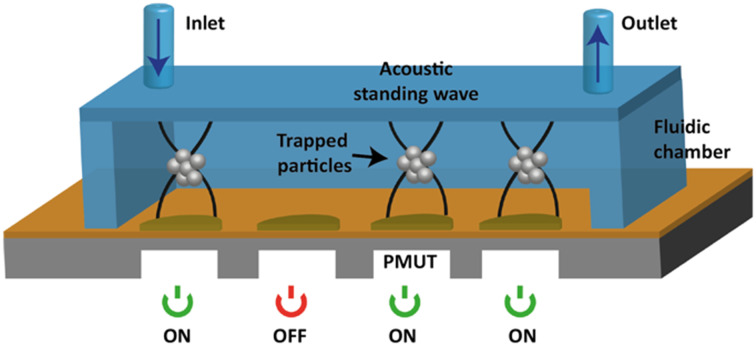

## Introduction

Acoustofluidic technologies leverage acoustic waves to manipulate a vast range of fluids and particles in a contactless fashion. These technologies offer several advantages over magnetic, optical, and optoelectronic micromanipulation techniques: unlike magnetic manipulation^1^ acoustic forces do not require magnetic labels, acoustic transducers operate at very low power,^2^ sound waves generate negligible heating compared to focused lasers,^3^ and manipulation can be performed in any aqueous environment without restrictions on ionic concentration.^4^ Acoustic manipulation is highly versatile and scalable, capable of manipulating particles ranging from nanometers to millimeters, within workspaces ranging from micrometers to centimeters,^5^ while performing tasks such as focusing,^6^ synthesizing,^7^ sorting^8^ and mixing.^9^ Consequently, acoustofluidic technologies have been considered for various biomedical applications such as single-cell manipulation, tissue engineering, medical diagnostics, and pharmacology.^10^

Acoustic micromanipulation is usually performed using a piezoelectric transducer that generates surface acoustic waves (SAWs) or bulk acoustic waves (BAWs). BAW transducers are typically macroscale piezoceramics such as lead zirconate titanate (PZT), sandwiched between two electrodes. The BAWs propagate through the entire fluidic volume, enabling the manipulation of particles away from the transducer up to a few millimeters.^11,12^ However, due to their composition, processing and bulk size, integrating piezoceramic transducers with microfluidic systems is challenging.^13^ On the other hand, SAW transducers consist of a piezoelectric substrate, such as lithium niobate (LiNbO_3_), with interdigital transducers (IDTs). SAW transducers are fabricated using conventional microfabrication techniques, allowing seamless miniaturization and integration with microfluidic channels and other microelectromechanical systems (MEMS) devices, such as sensors, to form lab-on-chip platforms. Additionally, microfabrication techniques enable mass production with high quality.

In SAW-based devices, standing waves are formed between one or several pairs of interdigital transducers (IDTs). Dynamic motion of particles or cells is achieved through various IDT designs, such as slanted-finger IDTs,^14^ chirped IDTs,^15^ or IDT arrays^16,17^ as well as through control strategies like phase shift^18^ and frequency modulation.^16,19^ The dynamic manipulation relies on the shift of the pressure nodes, which is defined by the fixed geometry of the IDTs, restraining the movement to a small workspace.^20^ Additionally, due to the periodic nature of standing waves, multiple acoustic traps are formed that cannot be moved independently, resulting in the collective motion of particles.^21^ Moreover, only a small percentage of the acoustic energy is transmitted to the fluid, with the majority of the energy confined to a layer approximately one wavelength thick above the substrate surface.^22^ Vertical motion of particles has only been demonstrated in a region up to 100 μm above the substrate.^18^ Recently, acoustic vortices have been developed to trap and move particles and cells in a larger workspace.^2,21^ Nevertheless, motion relies on the movement of the transducer relative to the fluidic chamber, requiring bulky motors and manipulators.

Piezoelectric micromachined ultrasound transducers (PMUTs) offer a compelling alternative to SAW-based systems, as they are microfabricated acoustic transducers that combine the generation of BAWs in fluid with seamless integration into lab-on-chip platforms. The generation of BAWs that propagate through the entire fluid volume enables micromanipulation of synthetic and biological samples well above the substrate.^23^ Furthermore, PMUTs are typically implemented as arrays of independently addressable transducers,^24^ each capable of generating a localized and tunable acoustic trap. Arrays can be designed with PMUT elements of varying resonant frequencies, geometries, and functionalities,^25^ enabling multiple PMUT types to coexist on a single chip for diverse acoustic operations and thereby offering greater flexibility than SAW-based systems.

PMUTs were initially developed for ultrasound imaging^24,26,27^ and communication applications,^28,29^ and only recently have they been investigated for broader biomedical applications.^30–32^ Consequently, despite their potential as acoustofluidic actuators, the use of PMUTs for particle manipulation remains largely underexplored. PMUT-based systems have so far been limited to manipulating particles near the transducer surface.^33–36^ To our knowledge, no studies have presented the integration of a PMUT array into a closed system to generate vertical standing waves for levitating and manipulating particles in a 3D aqueous space. Additionally, existing studies lack quantitative analyses of the generated acoustic pressure fields and resulting acoustic radiation forces.

In this article, we introduce an acoustic micromanipulation platform that is built around PMUTs for the deterministic and fine levitation and micromanipulation of particles in water. First, we present a PMUT array fabrication process that involves bonding a glass wafer to the array, facilitating its integration into a fluidic chamber. Then, we explored the ability of an array of PMUTs to selectively trap particles in pre-determined locations within a three-dimensional (3D) workspace through the formation of standing acoustic waves. Through a combination of experimental measurements and finite element (FE) simulation, we provide a comprehensive characterization of the acoustic field distribution and trapping dynamics. We demonstrate the dynamic transport and patterning of particles using a 4-PMUT array by selectively modulating the amplitude of the signals driving individual PMUTs. Finally, we showcase the integration of the PMUT array with fluidic systems and demonstrate its capability to manipulate particles under continuous flow.

## Results and discussion

### Device design and PMUT array fabrication

**Fig. 1:**
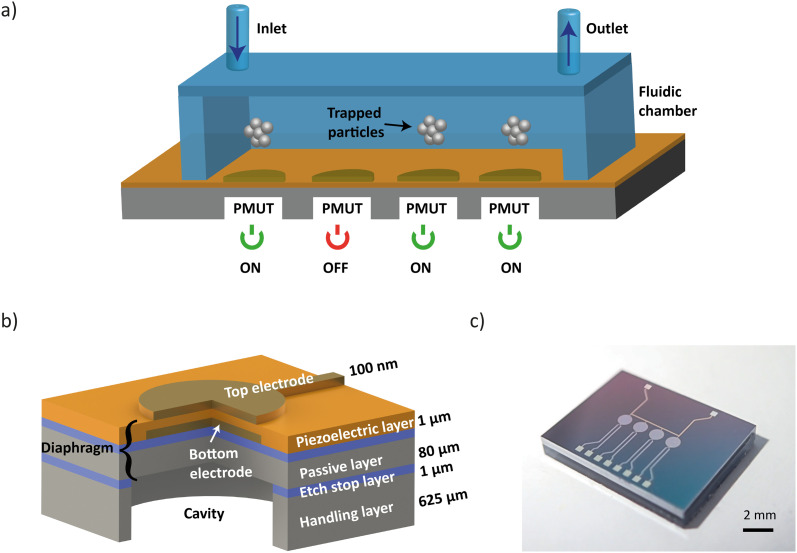
PMUT-based acoustic micromanipulation platform. a) Schematic illustration of the platform. Each PMUT element is independently addressable, enabling generation of localized bulk standing acoustic waves and acoustic traps for particle manipulation. b) Schematic showing the cross-sectional view a single PMUT element, illustrating electromechanically active and passive layers. c) Photograph of a 4-PMUT array.

The acoustic manipulation platform comprises a piezoelectric micromachined ultrasonic transducer (PMUT) array integrated with a fluidic chamber made of glass (Fig. 1a). The top glass surface functions as an acoustic reflector, enabling the formation of localized vertical standing acoustic waves within the chamber. To enable acoustic levitation of microparticles above the PMUT surface *via* bulk standing waves, the chamber height was set to approximately 1 mm. Assuming that the standing waves in the medium are in their first resonance mode (*i.e.*, *λ*/2 mode), the resonance frequency of the PMUTs should not exceed 700 kHz in water considering the fact that *λ* = *c*/*f* and the speed of sound in water is *c* = 1450 m s^−1^. Based on the known frequency range, the dimensions of the PMUTs can be selected accordingly. In this case, a radius of 450 μm and a passive thickness of 80 μm were found to meet the frequency requirement.

The diaphragm of a PMUT consists of a passive layer and a piezoelectric layer sandwiched between two metal electrodes (Fig. 1b). When an alternating current voltage is applied, the piezoelectric effect induces mechanical stress in the diaphragm, causing it to undergo periodic out-of-plane deflections, thereby generating vibrations. PMUTs are typically operated at their resonance frequency, where the diaphragm displacement is maximized.^37^ At this frequency, the vibration mode is characterized by a dome shape. The fundamental resonance frequency, *f*_0_, of the PMUT can be tailored through design, as it depends on the geometric parameters of the diaphragm and the intrinsic properties of the materials.^37^ Notably, intrinsic stresses introduced during the fabrication process can significantly affect the resonance frequency and the vibrational behavior of PMUTs. In plate mode, the vibroacoustic response of the diaphragm is governed by its flexural rigidity, *D*_e_, as shown in eqn (1). In membrane mode, the response is determined by the intrinsic pre-stresses, *T*_e_, as shown in eqn (2).^37^1
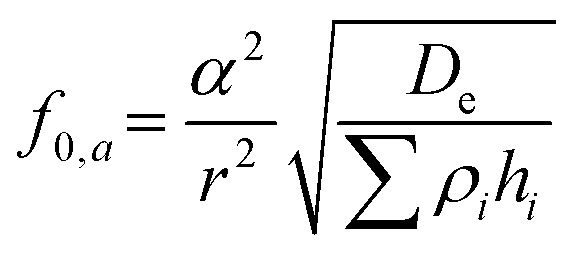
2
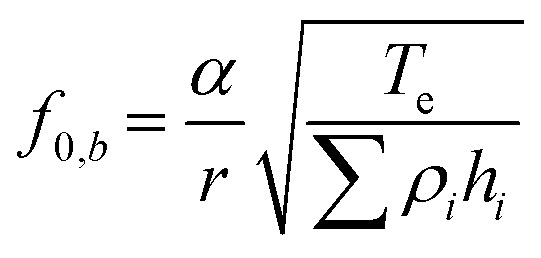
where *α* is the frequency parameter constant, *r* is the radius of the PMUT, and *ρ*_*i*_ and *h*_*i*_ are the density and thickness of the *i*th layer of diaphragm.

FE simulations based on eqn (1) can only provide an estimated range of resonance frequencies for a given PMUT design because they do not fully capture the mechanical effects of residual stress generated during microfabrication, which can significantly shift the resonance frequency. Therefore, experimental validation using laser Doppler vibrometer (LDV) is essential to accurately determine the resonance frequency and ensure reliable device performance.

**Fig. 2:**
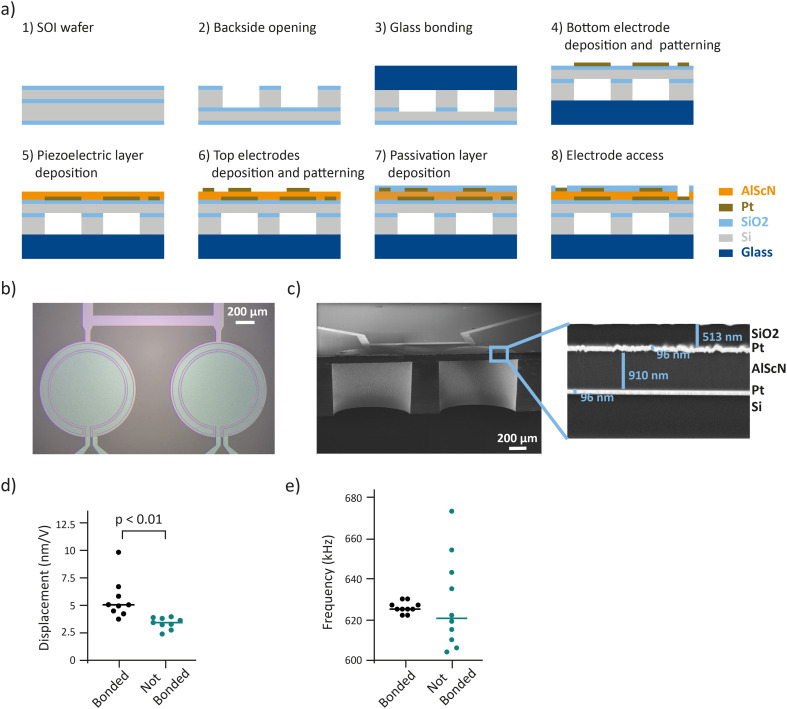
Fabrication and characterization of the transducer. a) Process flow diagram for the fabrication of PMUT arrays. b) Top view showing a 2-PMUT array. c) Cross-sectional electron microscopy image of a 2-PMUT array. The closeup view of the diaphragm shown on the right presents the thicknesses of the stacked thin-film layers. d and e) Comparison between PMUT arrays that are anodically bonded to glass (bonded) and without anodic bonding (not bonded). d) Displacement at the center of the PMUT measured at its fundamental resonance frequency. e) Resonance frequency of the PMUTs. All measurements were done in water on a PMUT array with radius = 450 μm and pitch = 1500 μm.

The PMUT array was fabricated using MEMS micromachining techniques and silicon on insulator (SOI) wafers (Fig. 1c). Aluminum scandium nitride (AlScN with a composition of Al_0.7_Sc_0.3_N) was used as the piezoelectric material because of its excellent electrochemical coupling, low-temperature deposition and ease of integration into the PMUT platform.^38^ The device layer of the SOI wafer defines the diaphragm thickness. Fig. 2a illustrates the main fabrication steps. Detailed information is provided in the Methods section. First, the backside of the oxidized SOI wafer was opened by deep reactive ion etching (DRIE) to create the cavities. Second, the oxide layer was removed by wet etching, followed by anodic bonding of a borosilicate wafer. Third, thin platinum electrodes (Ti/Pt, 10/100 nm) were sputtered on the device layer of the SOI wafer and patterned through lithography and dry etching. Fourth, a 1 μm-thick AlScN layer was sputtered, followed by deposition and patterning of the top platinum electrode (Ti/Pt, 10/100 nm). Fifth, a 500 μm-thick tetraethyl orthosilicate (TEOS) passivation layer was deposited to electrically insulate the top electrodes from the aqueous environment inside the fluidic chamber. Finally, access to the top and bottom electrodes was provided through successive wet etching steps. The PMUTs were fabricated on a 6-inch wafer, accommodating 115 arrays of various designs (Fig. S1a). More than 95% of the devices were functional. The common failure modes were defects in the electrode patterning and electrical insulation layer (Fig. S1b).

The PMUTs have a round shape, featuring a top electrode divided into a ring and a circle (Fig. 2b). This design promotes diaphragm displacement when a signal with a 180° phase shift is applied between the ring and the circle.^39^ The bottom electrodes that are interconnected through the tracks serve as a common ground. A representative scanning electron microscopy (SEM) image shows the cross-sectional view with the etched cavity and the various deposited layers (Fig. 2c). To achieve a high piezoelectric response, the AlScN crystals must grow perpendicular to the substrate. The crystallinity of the AlScN film was characterized using X-ray diffraction (XRD). Peaks corresponding to the wurtzite phase of AlScN(200), the face-centered cubic phase of Pt(111), and the face-centered cubic phase of Si (400) were identified (Fig. S2a). The AlScN film exhibits a fully *c*-axis textured structure with a clear (002) out-of-plane orientation. Additionally, the rocking curve of the (002) peak shows a full width at half maximum (FWHM) of 1.3° (Fig. S2b), indicating crystalline orientation with minimal defects.

A central feature in our fabrication process is the additional anodic bonding step between the SOI wafer and a 1 mm-thick borosilicate glass wafer. This bonding step, performed prior to AlScN deposition to avoid thermal damage to the piezoelectric layer, significantly enhances the mechanical stability of the diaphragm. The added structural rigidity provided by the bonded glass reduced parasitic vibrations and mechanical losses, resulting in improved displacement amplitude and greater reproducibility of the PMUT's fundamental resonance frequency in liquid environments (Fig. 2d and e). Ensuring good resonance frequency reproducibility after packaging is essential, as the formation of a standing wave requires a precise match between the fluidic chamber height and the PMUT resonance frequency. Therefore, anodic bonding facilitates the integration of PMUTs into confined aqueous environments such as lab-on-chip platforms, and increases the overall fabrication yield of the devices. Additionally, the increased diaphragm displacement enhances the functional capabilities of the device, enabling stronger acoustic output and more effective particle manipulation.

### Vibrational characterization

**Fig. 3:**
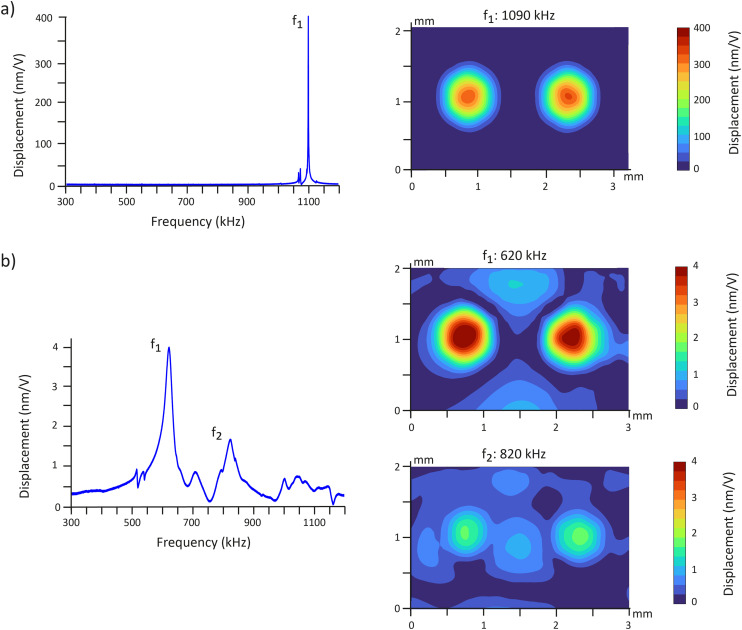
Vibrational characterization of a 2-PMUT array using a laser Doppler vibrometer. a) (Left) Frequency response of the 2-PMUT array measured in air with displacement recorded at the center of the left PMUT. (Right) Surface displacement map at the frequency *f*_1_, showing the spatial vibration profile as the maximum amplitude over all phases. b) (Left) Frequency response of the 2-PMUT array measured in water with displacement recorded at the center of the left PMUT. (Right) Surface displacement map at the frequency *f*_1_ and *f*_2_, showing the spatial vibration profile as the maximum amplitude over all phases.

The resonance frequencies and vibrational characteristics of a PMUT array comprising two transducers were measured using an LDV in both air and water. The LDV quantifies vibration amplitude and frequency by directing a laser beam at the vibrating surface and detecting the Doppler shift of the reflected light. In air, the PMUTs exhibited a clear resonance mode at 1090 kHz with a maximum displacement of the diaphragm of 410 ± 70 nm and a quality factor estimated to be around 900 (Fig. 3a). Surface scanning confirmed that the two PMUTs vibrate in phase in the fundamental mode at 1090 kHz, as confirmed by the dome-shaped deformation of the membrane. The fundamental resonance frequency is inversely proportional to the radius of PMUTs (Fig. S3a and b), consistent with membrane-mode behavior formulized by eqn (2). Furthermore, the maximum displacements were statistically similar across all radii (Fig. S3c), reinforcing this interpretation.^37^ The observed vibrational behavior, which matches the expected membrane-mode response, suggests the presence of significant intrinsic stresses that pre-bend the diaphragm and influence its vibroacoustic response.^37^ The center-to-center distance between PMUTs, referred to as pitch, has no impact on the resonance frequency or the maximum displacement (Fig. S3c and d).

The frequency response was more complex in water. Two distinct resonance peaks were identified at 620 kHz and 820 kHz, both exhibiting lower quality factors compared to those in air (Fig. 3b). The measured fundamental resonance frequency of 620 kHz is lower than the simulated value of 745 kHz for a PMUT of identical design (Fig. S4). We postulate that this discrepancy is stemming from intrinsic stresses introduced during fabrication, which can significantly affect the vibroacoustic response of the PMUT. PMUT arrays with different pitches exhibited similar frequency response spectra (Fig. S5a and b). The reduction of quality factor can be attributed to the viscosity of water, where viscous losses reduce the maximal displacement of the PMUTs by a factor of 80 relative to air (Fig. 3b). Additionally, the resonance frequency was lower due to the mass-loading effect of water. As the PMUTs vibrate, the water on their surface vibrates in tandem, effectively increasing the effective mass of the system and thereby lowering the resonance frequency. Surface scans revealed that the first peak corresponds to the fundamental mode, while the second peak corresponds to a damped version for a virtual PMUT of smaller radius. At 620 kHz, the PMUTs exhibit higher amplitude displacement and greater membrane movement across the surface compared to 820 kHz. Since the acoustic pressure generated in the chamber is proportional to the membrane displacement, operating at 620 kHz would produce stronger acoustic forces. Therefore, 620 kHz was selected over 820 kHz for the acoustic micromanipulation experiments to achieve more effective particle levitation and patterning. The maximum displacement of the PMUTs at 620 kHz was 5 ± 1.5 nm V^−1^ and the quality factor estimated to 20 (Fig. S5c). The displacement value was consistent among the PMUT arrays with different pitches. To ensure stable performance at higher actuation amplitude, we measured the PMUT response at elevated voltages. The data revealed a linear relationship between the input voltage and displacement up to 100 V (Fig. S6).

### Acoustic micromanipulation of particles in water

Based on the PMUT resonance frequency of 620 kHz, the chamber height should theoretically correspond to 1160 μm to support a vertical acoustic standing wave with a single pressure node. The measured height of the manufactured glass chamber was 1100 ± 100 μm. The experimental platform was assembled by mechanically clamping the glass chamber and a glass lid on top of the PMUT array (Fig. S7). The acoustic radiation force, *F*_rad_, acting on particles in a pressure field is derived from the Gor'kov potential, *U*_Gor_:^40^

3 *F*_rad_ = −∇*U*_Gor_

The potential function, *U*_Gor_, is defined as a function of the acoustic pressure *p* and the acoustic velocity *v*:4
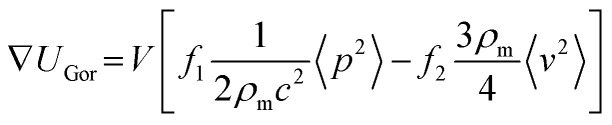
where *V* is the volume of the particle, *ρ*_m_ is the density of the fluid and *c* is the speed of sound of the fluid. The scattering coefficients are given by:5
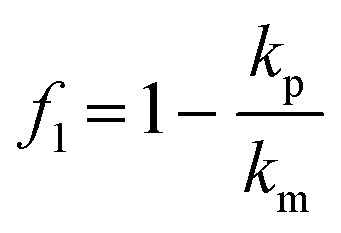
6
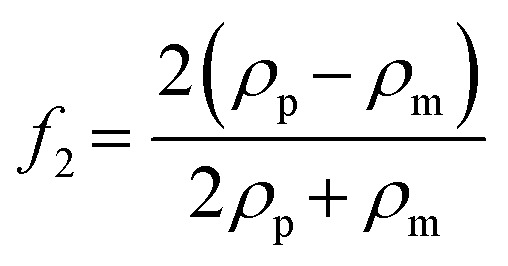
where *k*_p_ and *k*_m_ are the compressibilities of the particle and the fluid and *ρ*_m_ is the density of the particle.

FE simulations based on the Gor'kov theory were performed to investigate the mechanism of acoustic levitation with the PMUT array. The fluidic domain was modelled using impedance boundary conditions on the top and lateral surfaces to simulate the partial reflection and transmission of acoustic waves at the glass–fluid interfaces. The acoustic transduction of the PMUTs was implemented by interfacing the PMUT diaphragm with the fluidic domain. To recreate the vibration of the clamped diaphragm, a prescribed displacement was imposed to the center of the PMUT while its edges were mechanically fixed. Additionally, the movement of the silicon surface was implemented by adding a constant displacement boundary. The fixed displacement values were based on the LDV measurements. To simulate the particles movement, all relevant forces were incorporated, including gravitational force, buoyancy force, hydrodynamic drag, and the acoustic radiation force derived from the Gor'kov potential. All the parameters used in the simulations are described in detail in the Methods section.

**Fig. 4:**
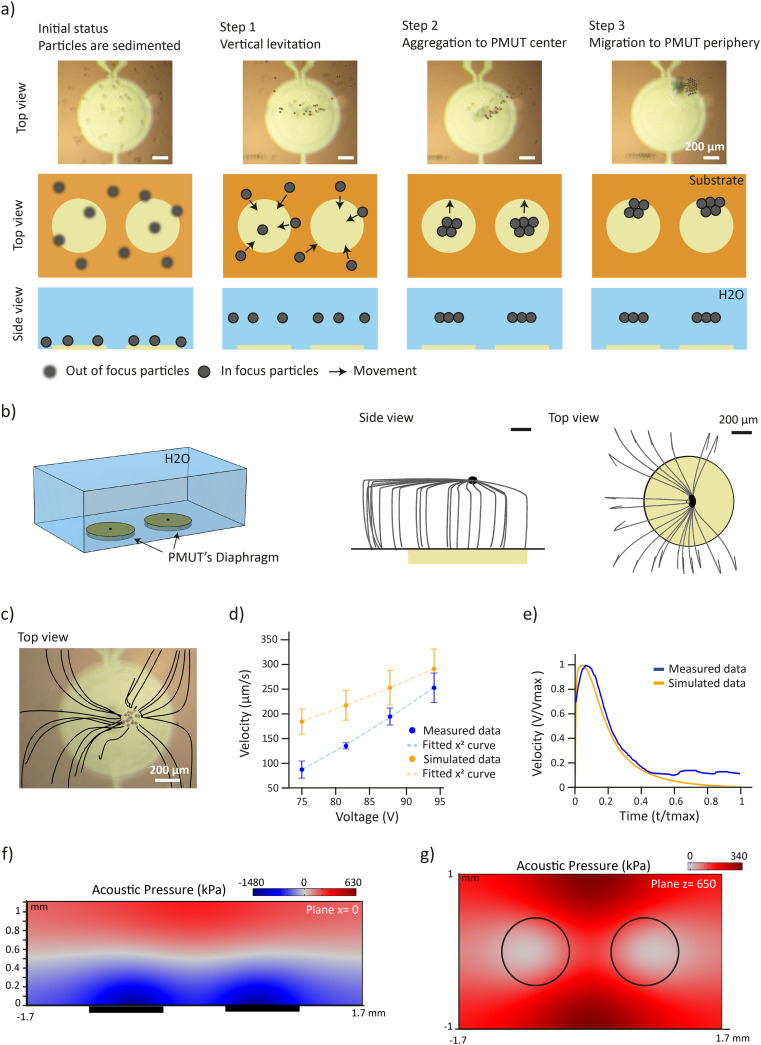
Dynamics of acoustic trapping of particles. a) Snapshots from Video S1 complemented with schematic illustrations showing the different steps of acoustic trapping. Step 0: particles sediment to the surface of the platform due to gravity. Step 1: upon activation of the PMUTs, particles are levitated. Step 2: particles migrate in-plane toward the center of the PMUT array and form aggregates. Step 3: particles migrate to the periphery of the PMUTs. b) (Left) Schematic representation of the FE model. (Right) Simulated particle trajectories shown from side and top views starting from the sedimented state. c) Measured trajectories of particles during step 2 (see Video S4). The trajectories are overlaid on the picture as black lines. d) Maximum in-plane particle velocities during step 2 as a function of excitation voltage. Simulated data represent the averaged maximum velocity across 20 different trajectories. e) Representative velocity profiles over time of a single particle from experimental and simulated data. f) Simulated acoustic pressure distribution in the cross-sectional plane at *x* = 0 μm at 75 V_p_ excitation. Dark rectangles indicate PMUT locations. Regions of zero pressure are shown in grey. g) Simulated pressure field in the levitation plane at *x* = 640 μm at 75 V_p_ excitation. Dark circles indicate PMUT locations. Regions of zero pressure are shown in grey.

Upon excitation at 620 kHz, polystyrene (PS) particles with diameters ranging from 15 μm to 100 μm were successfully levitated and trapped on top of the PMUTs (Fig. 4a and S8, Videos S1 and S2). All subsequent analyses in this study were performed using 30 μm diameter PS particles. The levitation was confirmed by immobilizing the PS particles in a transparent hydrogel. The PS particles were manipulated in the hydrogel precursor solution, and while the particles were trapped by the acoustic waves, the hydrogel was crosslinked by UV exposure (Fig. S9a). The levitation height was measured as 630 ± 30 μm (*n* = 12) by analyzing the cross-section of the hydrogel (Fig. S9b and c). Experiments were conducted on three PMUT arrays with twelve samples in total (four per array).

The trapping can be divided into 3 steps (Fig. 4a and Video S1). First, the particles were levitated from the bottom surface of the chamber to the plane prescribed by the standing wave. Second, the particles were attracted to a region above the center of the PMUT. Third, the particles occasionally migrated to a region that is above the periphery of the PMUT. FE model recapitulated the first two steps of particle movement, but the particles stabilized in the middle region in simulations unlike the experimental observations (Fig. 4b and Video S3). The trajectories and velocities of the particles were measured from the in-plane movement of the particles at different voltages (Fig. 4c and Video S4) which were then compared with the simulation results. Simulations showed that particles coming from different directions had slight differences in their velocity (Fig. S10a). To capture this effect, the simulated data are based on the average and standard deviation of 20 particles coming from the surroundings of the PMUT. Both simulated and experimental velocities are in the same order of magnitude and follow a similar trend: the velocity increases quadratically with the voltage (Fig. 4d and S10b). This quadratic dependence of the acoustic radiation force on voltage is characteristic of a standing field.^40^ Since our fluidic chamber is enclosed by reflective walls, a stationary pressure pattern naturally forms, which can be assimilated to a standing wave. The slight discrepancy between the simulated and measured velocities can be attributed to several factors. First, although the computational model was designed to closely replicate the experimental setup, the values of some key parameters such as the acoustic impedance of the glass and the physical properties of the polystyrene particles may slightly differ from their actual values. Additionally, the experimental data were collected using four different PMUT arrays, each exhibiting small variation in their displacement performance (Fig. 2d). Finally, in the physical system, energy losses may occur through mechanisms not accounted for in the simulation, potentially explaining the higher velocities predicted by the model.

Considering a steady movement of a particle at low Reynolds number, the force acting on a particle can be derived from its velocity, *v*, using the Stokes drag:

7 *F*_rad_ = *F*_drag_ = 6π*Rηv*

where *R* is the particle radius and *η* is the fluid dynamic viscosity. Using eqn (7), the planar acoustic force was calculated to be 25 pN at peak voltage of 75 V_p_. The reported driving voltages are always reported as peak voltages (V_p_). Direct calculation of the planar acoustic radiation force and drag force in the simulations confirmed this force value (Fig. S10c), validating the use of the Stokes drag. Both experimental and simulation data showed that the particle velocity initially increases to a peak before decreasing as the particle approaches the center of the PMUTs (Fig. 4e).

These results demonstrated that the FE model recapitulates the particle dynamics. Consequently, other variables of interest that cannot be directly measured such as pressure can be extracted from simulations. The vertical acoustic force was estimated to be 800 pN at 75 V_p_ (Fig. S10d). Notably, the acoustic force significantly exceeds the gravitational force acting on a PS particle, *F*_g_ = 145 pN, demonstrating that acoustic levitation is feasible under these conditions. The movement of the particles in the first two trapping steps can be explained by the high difference between the vertical and in-plane acoustic force. Additionally, the particles were levitated to a distance *h* = 640 μm above the PMUTs (Fig. S10e), which is in very good agreement with the measured values (Fig. S9c). Moreover, the vertical pressure distribution confirmed the presence of a standing wave above the PMUTs (Fig. 4f). The maximum pressure in the chamber was estimated to be 1.5 MPa. At the levitation plane, the pressure directly above the PMUTs was 0 while the maximum pressure was computed as 350 kPa close to the wall (Fig. 4g), confirming the formation of acoustic traps. Finally, the simulation can be used to explore alternative platform designs and manipulation patterns. For instance, by tuning the chamber height, the platform could be extended to generate multiple acoustic nodes, enabling simultaneous multi-plane trapping (Fig. S11a and b).

Particles may experience dielectrophoretic forces due to the electric field, or drag forces induced by acoustic streaming. Although these effects were not observed experimentally, we performed simulations to assess their potential impact. First, electric field simulations indicated that at an actuation voltage of 75 V_p_, the electric field generated within the fluidic chamber is on the order of 10^−4^ V m^−1^ (Fig. S12). This magnitude is significantly lower than the typical electric field strengths required to manipulate small particles, which generally range from 10^3^ to 10^5^ V m^−1^.^41,42^ Furthermore, the electric field is primarily concentrated near the surface of the PMUTs. Since experimental observations show that the particles levitate rather than remain adhered to the PMUT surface, we conclude that electrical forces are negligible in this context. Second, fluid dynamics simulations confirmed the presence of acoustic streaming within the chamber, with maximum flow velocities localized near the PMUT surface (Fig. S13a). Particle tracing simulations demonstrated that, for PS particles with a diameter of 30 μm, the induced acoustic streaming alone is insufficient to influence particle motion (Fig. S13b). Consequently, acoustic streaming does not contribute to the observed particle trajectories or acoustic trapping behavior (Fig. S13c).

### Influence of chamber height on trap dynamics

**Fig. 5:**
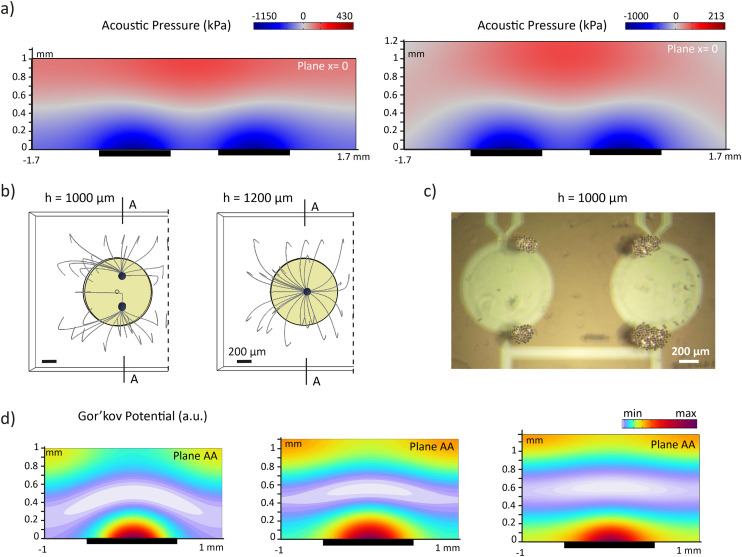
Influence of chamber height on particle motion. a) Simulated acoustic pressure distribution in the vertical cross-sectional plane at *x* = 0 μm at 75 V_p_ excitation for two different chamber heights: *h* = 1000 μm (left) and *h* = 1200 μm (right). Dark rectangles indicate PMUT locations. Regions of zero pressure are shown in grey. b) Simulated top-view trajectories of particles starting from a sedimented state for *h* = 1000 μm (left) and *h* = 1200 μm (right). c) Snapshots from Video S5 showing levitated PS particles at *h* = 1000 μm. d) Simulated Gor'kov potential along the cross-sectional plane AA (as defined in (b)) for chamber heights of *h* = 1000 μm (left), *h* = 1100 μm (middle), and *h* = 1200 μm (right), illustrating the impact of chamber height on Gor'kov potential distribution. Dark rectangles indicate PMUT locations.

To create an ideal standing wave with a single node, the chamber height should be equal to half the wavelength of the acoustic wave. However, manufacturing and assembling the glass chamber with high precision is challenging, and tolerances may result in deviations that cannot be neglected. In our platform, the clamping system ensures a reliable assembly without altering the effective liquid height. However, the manufacturing of the glass chamber by machining introduces a height tolerance of ±100 μm, for a total liquid height of 1000 ± 100 μm. To quantify the sensitivity of the trapping behavior to the liquid height deviations, we employed FE simulations to study the effect of chamber height mismatches within ±100 μm. In both cases, a standing wave forms above the PMUTs, similar to the situation at 1100 μm (Fig. 5a). Interestingly, simulations revealed that in a chamber with *h* = 1200 μm, particles are still trapped at the center of the PMUTs, while with *h* = 1000 μm, they are trapped in two different regions above the PMUTs (Fig. 5b). These two traps are positioned along the *x*-axis towards the edges of the PMUTs while maintaining a similar *y*-axis position as the single trap. Experimental observations confirmed the formation of dual trapping sites at *h* = 1000 μm (Fig. 5c and Video S5). Analyzing the Gor'kov potential revealed that the regions of minimum Gor'kov potential were spread across the PMUTs' surface. Along the AA plane, the Gor'kov minimum has a parabolic shape at a chamber height of 1000 μm and a disc shape at 1100 μm and 1200 μm (Fig. 5d). Along the plane *x* = 0, the Gor'kov minima are similar in all conditions and have the shape of a disc (Fig. S14).

Since the acoustic radiation force is the derivative of the Gor'kov potential, regions with a low Gor'kov gradient experience minimal force. Therefore, within the Gor'kov minimum, the in-plane or vertical acoustic force is expected to be nearly zero. Nevertheless, particles are still subjected to gravity and thus settle in the regions of the Gor'kov minimum with the lowest height. At chamber heights of 1200 μm and 1100 μm, particles are stable at their initial position due to the flat disc shape of the Gor'kov potential, resulting in a single aggregate. At a reduced chamber height of 1000 μm, the paraboloid landscape creates two stable minima, leading particles to settle at these energetically favorable positions and form two distinct aggregates of particles. In conclusion, the variation in glass chamber height introduced by machining does not prevent the levitation and trapping of PS particles, but may influence the position and number of trapping sites. To minimize these effects, the chamber height should be more precisely controlled, for example by performing glass polishing with higher accuracy.

In the third step of levitation (Fig. 4a), the particles consistently migrated toward the edges of the PMUTs, corresponding to the locations of two distinct traps observed when *h* = 1000 μm. Based on computational analysis, we hypothesize that this behavior is linked to the shape of the Gor'kov potential. Specifically, when the chamber height is slightly below 1100 μm, simulations show that the Gor'kov potential adopts a parabolic profile, making the center of the PMUT an unstable equilibrium point. As a result, even minor perturbations can cause the particle aggregate to shift toward the stable equilibrium positions at the edges of the PMUT. While the clamping system ensures that no additional height is introduced to the platform, the glass chambers have a height tolerance of ±100 μm, which may account for the observed deviation from ideal trapping at the center. Due to the migration of the PS particles to the PMUTs periphery, the precision of the horizontal trapping is defined by the PMUTs radius. One possible solution to achieve more precise control over particle positioning is to reduce the size of the PMUTs, thereby increasing spatial confinement and making the Gor'kov potential profile more localized. Another potential solution is to reorient the system by placing the PMUT array on top. In this configuration, the stable equilibrium would shift to the center of each PMUT, as the periphery of the Gor'kov potential would lie at a higher position within the fluid.

### In flow particle trapping

**Fig. 6:**
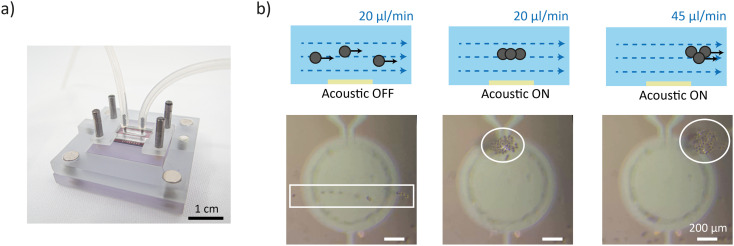
In-flow acoustic particle manipulation. a) Photograph of the fluidic chip mounted on top of the PMUT array. b) Snapshots from Videos S6 and S7 describing the acoustic trapping within an applied flow. (Left) In the absence of acoustic activation, particles follow the fluid streamlines at a flow rate of 20 μL min^−1^. (Middle) Upon activation of the PMUT array, particles are acoustically trapped at the nodes of the bulk standing acoustic wave above the transducers. (Right) At an increased flow rate of 45 μL min^−1^, the hydrodynamic force exceeds the acoustic radiation force, causing particles to escape from the trap and move along the streamlines.

Following the establishment of localized acoustic traps above the PMUT array, we investigated the capacity of the system to retain particles under continuous fluid flow. To this end, the experimental setup was modified to include an inlet and an outlet integrated into the top of the fluidic chamber, enabling connection to a peristaltic pump for controlled flow (Fig. 6a). In this configuration, PS particles were introduced as a suspension to the chamber by the imposed flow (Fig. 6b and Video S6). In the absence of acoustic excitation, the particles followed the streamlines of the laminar flow, passing unhindered over the PMUT surface at a nominal flow rate of 20 μL min^−1^. Upon activation of the PMUT array standing acoustic waves were established, and incoming particles were effectively captured within the acoustic potential wells. The observed particle behavior under flow mirrored that in static conditions. Initially, individual particles were trapped above the center of each PMUT and subsequently migrated toward the periphery, where they formed stable aggregates.

The magnitude of the drag force imposed by the flow was estimated using Stokes' law. At 20 μL min^−1^, the drag force was calculated to be approximately 45 pN. To counteract this, the PMUTs were actuated at 88 V, generating an in-plane acoustic radiation force of 55 pN sufficient to overcome the hydrodynamic drag, thereby maintaining particle trapping. To assess the performance limits of the acoustic trapping under flow, we increased the driving voltage to 100 V, generating an acoustic radiation force of 90 pN, and incrementally increased the flow rate (Video S7). Particles remained stably trapped up to a threshold flow rate of 40 μL min^−1^. At 40 μL min^−1^, the hydrodynamic drag force is expected to be the same as the acoustic radiation force. While trapping was still effective, the particle aggregates became visibly more diffuse, indicating partial compromise of the confinement. At a flow rate of 45 μL min^−1^, the acoustic force was no longer sufficient to retain the particles, which started to flow along the streamlines (Fig. 6b and Video S7). These results highlight the robust performance of the PMUT-based acoustic platform for particle manipulation under low-to-moderate flow conditions and demonstrate the potential of this system for applications that require in flow micromanipulation.

### Selective particle trapping and dynamic manipulation

**Fig. 7:**
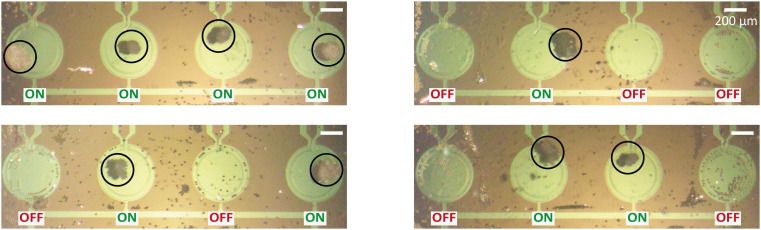
Selective acoustic trapping using a 4-element PMUT array. Individual PMUTs in a 4-PMUT array are turned on and off to selectively trap PS particles on the active units and aggregate the particles into the desired patterns.

To further explore the practical applications of the PMUT-based acoustic micromanipulation system, we investigated the dynamic positioning of PS particles into predefined patterns using a 4-PMUT array. By selectively controlling the on/off states of individual PMUTs, we were able to levitate and trap PS particles on the active PMUT units (Fig. 7). Dynamic manipulation of the signal amplitude enabled the generation of moving particle patterns within the array.

**Fig. 8:**
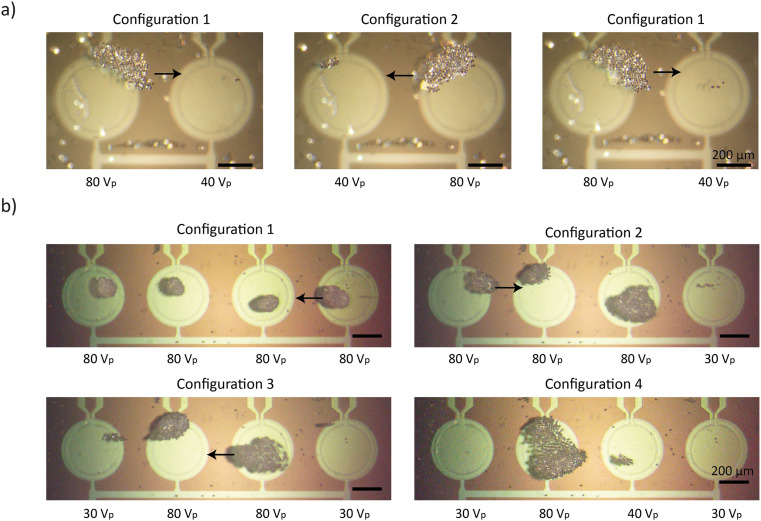
Translation and merging of PS particles aggregates using PMUT-based acoustic control. a) Snapshots from Video S8 demonstrating the controlled lateral translation of a PS particle aggregate from the left to the right PMUT. Arrows indicate the direction of motion. b) Snapshots from Video S9 showing the translation and merging of multiple particle aggregates. Configuration 1: initial state with four distinct aggregates. Configuration 2: the fourth aggregate is translated and merged with the third one. Configuration 3: the first aggregate is translated and merged with the second one. Configuration 4: the third aggregate is translated and merged with the second one, bringing all the particles together.

In the first set of experiments, we demonstrated dynamic particle translation between adjacent PMUTs by modulating the voltage amplitude applied to each unit (Fig. 8a and Video S8). Specifically, both PMUTs were driven with continuous sinusoidal signals at the same frequency (620 kHz) and with the same phase, but with differing amplitudes: one PMUT operated at full voltage (80 V_p_) while the adjacent unit was driven at 50% of that amplitude (40 V_p_). This amplitude differential created an imbalance in the acoustic radiation force, weakening the trap above the lower-voltage PMUT and enabling particles to migrate toward the stronger trap. The translation occurred immediately upon switching the signal amplitudes, with no noticeable delay or transient behavior, indicating a rapid response of the system to the control input. By alternating the voltage levels between the two PMUTs, we achieved controlled back-and-forth translation of particle aggregates. In the second set of experiments, we applied this differential control strategy to a 4-PMUT array to guide particles toward a central location and merge them into a single aggregate (Fig. 8b and Video S9). Initially, all four PMUTs were driven with identical signals (620 kHz, 80 V_p_, in-phase). Then, the edge PMUTs (PMUT 1 and 4) were reduced by 60% to 30 V_p_, causing particles to migrate toward the central PMUTs (PMUT 2 and 3). Finally, the voltage on PMUT 3 was reduced by 50% to 40 V_pp_, resulting in the final aggregation of particles at PMUT 2. The merged particle aggregates can be split into sub-groups by controlling the individual PMUTs (Video S10). These results demonstrate that by precisely tuning the relative amplitudes of the driving signals, we can dynamically reconfigure the acoustic potential landscape and achieve programmable, directional particle transport.

## Conclusion and outlook

We presented a PMUT-based acoustic platform for the deterministic levitation and manipulation of particles within a closed fluidic cavity. The system operates by generating vertical acoustic standing waves, which relies on a precise match between the PMUT resonance frequency and the chamber height. To improve frequency stability and device yield, we modified the standard PMUT fabrication process by bonding a thick glass layer to the PMUT array, enabling more reliable resonance characteristics. In addition, we developed a 3D FE model of the platform to characterize both the acoustic pressure and streaming fields. Using this model, we performed a detailed analysis of the acoustic forces acting on particles, offering quantitative insight into the underlying trapping mechanisms. Finally, we demonstrated the platform's ability to trap particles in flow and dynamically reconfigure acoustic traps, highlighting its versatility for advanced acoustofluidic applications. Collectively, these contributions represent a significant advancement in PMUT-based acoustofluidics, particularly for applications demanding precise and volumetric particle control within closed fluidic environments such as cell and organoid sorting or assembly. In our future work, we plan to miniaturize the PMUT elements and fabricating denser *n* × *n* arrays to enhance the spatial resolution of acoustic manipulation. Additionally, phased array control will be implemented to shape and steer acoustic fields by independently modulating the phase and amplitude of each transducer. This approach would enable the generation of highly customizable acoustic fields, allowing for precise 3D positioning and dynamic movement of particles along arbitrary trajectories.^43–45^ Finally, in the future, we envision the platform being applied to life science research, particularly for cell and organoid manipulation and tissue engineering.

## Methods

### Fabrication of PMUT array

The PMUTs were fabricated from a 150 mm SOI wafer (Ultrasil) that consists of an 80 μm-thick silicon (Si) device layer, 1 μm-thick buried silicon oxide (BOX) layer, and a 625 μm-thick (100) silicon handle substrate. A 1 μm-thick oxide was grown on both sides of the wafer. The backside of the oxidized SOI wafer was etched by DRIE (SPTS, Rapier) to create cavities. Then, the oxide layer was removed by a buffered hydrofluoric acid (BHF) 7 : 1 wet etching. A 150 mm Borofloat 1 mm-thick wafer (Plan Optik) was anodically bonded to the back side of the SOI wafer (EVG520is bonder). The wafer was then flipped, and platinum electrodes (Pt/Ti, 100/10 nm) were sputtered (LLS2, Evatec) and patterned through lithography and dry etching. Afterwards, a 1 μm-thick AlScN layer was sputtered (Clusterline 200, Evatec) in reactive mode from an AlSc compound target. The top platinum electrode (Pt/Ti, 100/10 nm) was then sputtered and patterned as the bottom electrode, followed by the plasma-enhanced chemical vapor deposition of a 500 nm-thick TEOS oxide (SPTS APM). Access to the bottom electrode was created by successive BHF 7 : 1 and KOH 40% at 60 °C wet etching. Finally, access to the bottom electrode was created by a final BHF wet etching. The individual dies were released by dicing (DAD321, Disco). A resinous blade with an inner diameter of 51 mm and an outer diameter of 56 mm (R07-SD400-BB200-85, Disco) was rotated at 25 000 rpm and translated at a feed rate of 1 mm s^−1^.

### Fabrication of glass chamber

The glass chamber was fabricated using computer numerical control (CNC) machining (Verre and Quartz Technique SA). A borosilicate sheet was first polished to the desired height, after which a CNC process was used to cut the sheet to the required dimensions and create a central hole. The glass lid was fabricated from a 1 mm borosilicate glass wafer, which was diced to the appropriate size using the same parameters as before. For experiments requiring fluid flow, small inlet and outlet holes were drilled on the lid using CNC machining.

### Imaging

The cross-section image of the PMUT array was taken using a SEM (Zeiss Crossbeam 550, Carl Zeiss AG). The sample was diced in its middle with a wafer dicer as described before. The diced samples were mounted on aluminum stubs using conductive carbon tape without any additional conductive coating. Imaging was carried out at an operating voltage of 5 kV and a beam current of 100 pA, with a working distance of 4 mm. The zoom image of the deposited layers was taken with another SEM (Scios 2, Thermofisher). First, a 20 nm-thick conductor gold layer and a 1 μm-thick protective platinum were deposited. A focused ion beam (FIB) was used to create a 20 × 20 μm^2^ cavity with a depth of 10 μm. Images were taken with high resolution secondary electron detector in chemical contrast mode at an operating voltage of 5 kV and a beam current of 100 pA.

### XRD characterization

XRD measurements were performed using a materials research diffractometer (Panalytical X'Pert) with Cu Kα radiation of wavelength 1.5406 Å. The diffraction patterns were recorded over a 2*θ* range of 30° to 80° with a step of 0.03° at a scanning speed of 1 s per step. The rocking curve of the AlScN peaks was recorded over an *ω* range of 10° to 25° with similar parameters as the above.

### Vibrational characterization

An LDV (NLV-2500, Polytech) was used to measure the surface vibrations in air and in water. A periodic chirp signal with a peak voltage (V_p_) amplitude of 3 V_p_ was applied for frequencies between 300 kHz and 1200 kHz. The surface was scanned using the motorized stage of the LDV with a grid density of 15 points per mm^2^. The frequency response spectrum was measured at the middle of the left PMUT. The displacement is reported as V nm^−1^ which corresponds to the maximum displacement from the rest position divided by the applied peak voltage. To be consistent, all the voltages are reported as peak voltage (V_p_). The measurements in water were performed with the exact same setup as the manipulation experiment. A custom clamping setup was designed and 3D printed to position the glass chamber on the PMUT array and make the electrical connection (Fig. S7). The clamping setup consists of a substrate, two chamber guides, a clamping piece and a PogoPin holder. The substrate has a compartment to hold the PMUT array and four pins to ensure good alignment of the different components. Two guides are inserted on the pins to ensure the precise positioning of the chamber. The chamber is magnetically clamped using a clamping piece. Finally, the PogoPin holder is inserted on the pins and magnetically maintained to create electrical contact with the PMUT array. The PogoPin holder also fulfills the role of clamping the glass lid on the glass chamber. The measurements in air were performed without the glass chamber. For the linearity measurements (Fig. S6), displacement was recorded at a frequency slightly offset from the resonance frequency. This allowed the applied voltage to be increased up to 100 V_p_ while ensuring that the measured displacement remained below the instrument's upper limit of 140 nm.

### Acoustic manipulation of particles in water

The experiments were performed using the same clamping setup as for the vibrational characterization. PS particles with a diameter of 30 μm (84135, Sigma) were suspended in deionized water with a volume ratio varying from 1 : 100 to 1 : 10. Acoustic waves were generated by the application of a sinusoidal signal (75 V_p_ and 620 kHz) using a wave generator (33120A, Hewlett Packard) and a voltage amplifier (WMA-300, Falco Systems). All the PMUTs were simultaneously activated with an in-phase signal when not specified otherwise. Levitation videos (Videos S1, S2 and S5) were recorded using a CMOS camera (uEye, IDS Imaging Development Systems GmbH) mounted on a stereomicroscope (Olympus SZX16). For the dynamic translation and merging of PS particles, all the PMUTs were simultaneously activated with an in-phase signal generated using a 4-channel wave generator (Moku:Pro, Liquid instrument) and two 2-channel voltage amplifier (2350, TEGAM).

### Acoustic manipulation of particles in hydrogel precursor

The poly(ethylene glycol) diacrylate (PEGDA) hydrogel precursor solution was prepared by mixing 8 v% PEGDA (Mw = 700, Sigma Aldrich, #455008) and 0.05 wt% LAP (Sigma Aldrich, #900889) in deionized water. PS particles with a diameter of 30 μm were suspended in the PEGDA hydrogel precursor solution at a volume ratio of 1 : 100. Acoustic waves were generated by applying a sinusoidal signal (75 V_p_, 620 kHz) using a wave generator (33120A, Hewlett Packard) and a voltage amplifier (WMA-300, Falco Systems). The signal was applied for 10 s, followed by UV exposure while maintaining the acoustic signal. The hydrogel was exposed to 365 nm UV light (Panasonic, UJ30/35 Series) for 15 s at an intensity of 590 mW cm^−2^ (total illumination dose = 8.9 J cm^−2^) to achieve crosslinking. Once crosslinked, the hydrogel was retrieved from the platform and placed on a glass slide to image the *xz* plane using a 5× objective (Epiplan, 442920, NA = 0.13) mounted on an inverted microscope (Primo Vert, Zeiss). The height *H* was measured using FIJI^46^ software by determining the distance between the surface in contact with the PMUT and the center of the particle aggregate.

### Acoustic trapping in flow

For the demonstration of in-flow trapping, the experimental setup was modified to include metallic fluidic inlet and outlet ports (inner diameter 0.5 mm, outer diameter 0.8 mm, AlSl 304, Unimed) integrated into the top of the glass chamber. These ports were connected to silicone tubing (inner diameter 0.5 mm, outer diameter 1 mm, 3100525, Eur.Pharm) and linked to a peristaltic pump (ISM936D, Ismatec). The PS particles were suspended in deionized water at a volume ratio of 1 : 50 and introduced into the chamber *via* the inlet tubing. Flow rates were varied from 20 μL min^−1^ to 45 μL min^−1^ to assess the limits of the acoustic platform. Acoustic waves were generated by the application of a sinusoidal signal (88 V_p_ to 100 V_p_ at 620 kHz) using a wave generator (33120A, Hewlett Packard) and a voltage amplifier (WMA-300, Falco Systems). The videos were recorded with a CCD camera (acA1920-155um, Basler) mounted on a stereomicroscope (Olympus SZX16).

### Particle tracking

Videos were processed with Fiji^46^ and then analyzed using the TrackMate plugin.^47^ First, the video was cropped to keep only the interesting frames. Second, the background was subtracted with the option “light background” and “create background” ticked and a rolling ball radius of 50 pixels. Third, the video type was changed to 8-bit, and the video was segmented by adjusting the threshold manually. The same threshold was applied to all the frames. Fourth, the binary functions “close” and “fill holes” were used to close the particles' shapes. Fifth, the TrackMate plugin was applied with a thresholding detector and an advanced Kalman tracker. As the maximum velocity of the particles depends on their starting position, we considered for our calculation only the particles coming out of the frame. The velocity *vs.* time curve was extracted for each particle. The maximum velocities were calculated as the average maximum velocity ± standard deviation. We measured the particle velocity at four different voltages 75 V_p_, 81 V_p_, 87 V_p_ and 93 V_p_ for three PMUT array. For each voltage, three to four videos were recorded for a minimum of 12 particles analyzed.

### Finite element simulation

The software COMSOL Multiphysics 6.3 was used for the FE simulations of the acoustic platform. The model consisted of three domains: the water domain and the two simplified PMUT's diaphragms made of an 80 μm-thick silicon layer and 1 μm-thick AlScN layer. The “pressure acoustic, frequency domain” module was implemented to the water domain and the “solid mechanic domain” to the two diaphragms. The acoustic transduction of the PMUTs was implemented by interfacing the PMUT's diaphragm with the fluidic domain through the multiphysics “acoustic-structure”. A prescribed displacement of 5 nm V^−1^ was imposed to a 50 μm diameter circle at the middle of each PMUT while the edge of the PMUTs were fixed to mimic a clamped diaphragm. The displacement value was taken as the average maximum displacement of the PMUTs (Fig. 2d). For the water domain, an acoustic impedance boundary of 13 MPa s m^−1^ was set for the side and top walls. To the bottom of the water domain, a prescribed acoustic displacement of 0.3 nm V^−1^ was applied to mimic the vibration of the silicon substrate. The displacement value was taken as the average displacement from the silicon substrate calculated form the LDV scan at 620 kHz (Fig. 3b). The Gor'kov potential was calculated from the total acoustic pressure. The relevant formulas and constants are provided in Table S1. For all the simulations, the simulations were run with a frequency of 620 kHz and a driving voltage of 75 V_p_ when not specified otherwise. To estimate the trajectories of particle and the forces applied to them the module “particle tracing for fluid flow” was used. The acoustic force, the gravity, the buoyancy and the drag force were applied on spherical objects (diameter = 30 μm and density = 1100 kg m^−3^). The acoustic force in the module is defined following the Gor'kov theory. To recreate the condition close to the experimental data acquired for the particle tracking experiments, 20 particles were released at the bottom surface on the edge of a 750 × 550 μm^2^ rectangle centered around the left PMUT. For each particle, the in-plane velocity was calculated as the amplitude of the in-plane velocity vector from 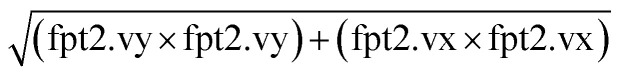. The maximum velocities were then calculated as the average maximum velocity ± standard deviation of the 20 particles. The acoustic streaming inside the platform was studied by implementing the physic module “laminar fluid flow” and the multiphysics modules “acoustic streaming domain coupling” and “acoustic streaming boundary coupling”. The electric field was studied by implementing the “electrostatic” physics module.

## Author contributions

E. V.-D.-B. designed the PMUTs array, developed the acoustic platform, designed and performed all the LDV characterization experiments, manipulation experiments, and numerical simulations under the supervision of G. W., M. D., and M. S. S. M.-A. D. and T. O. developed the fabrication process and fabricated the PMUT array. E. V.-D.-B. and J. H. developed the protocol for particle tracing and analyzed the data. D. B. provided technical advice on FE simulations. D. B., M.-A. D., T. O., S. H. and A. D. provided technical advice on platform development and experiment designs. E. V.-D.-B., M. D. and M. S. S. interpreted the data and wrote the manuscript. All the authors reviewed the manuscript.

## Conflicts of interest

There are no conflicts to declare.

## Supplementary Material

LC-025-D5LC00747J-s001

LC-025-D5LC00747J-s002

LC-025-D5LC00747J-s003

LC-025-D5LC00747J-s004

LC-025-D5LC00747J-s005

LC-025-D5LC00747J-s006

LC-025-D5LC00747J-s007

LC-025-D5LC00747J-s008

LC-025-D5LC00747J-s009

LC-025-D5LC00747J-s010

LC-025-D5LC00747J-s011

## Data Availability

Supplementary information: SI and videos are available as separate PDF and MP4 files. See DOI: https://doi.org/10.1039/D5LC00747J. The data supporting this article have been included as part of the article or the supplementary information (SI). The detailed datasets generated and analyzed during the current study are available from the corresponding authors on reasonable request.
