# Supplementary material for: On-chip particle levitation and micromanipulation using bulk acoustic waves
Source: Lab Chip. 2025 Oct 2;25(22):5961–75. doi: 10.1039/d5lc00747j (PMC12512227; doi:10.1039/d5lc00747j)
Supplement: LC-025-D5LC00747J-s001 [file LC-025-D5LC00747J-s001.pdf]

Supplementary Information (SI) for Lab on a Chip.
This journal is © The Royal Society of Chemistry 2025

Supplementary Information

On-chip particle levitation and micromanipulation using bulk acoustic waves

Emilie Vuille-dit-Bille, Marc-Alexandre Dubois, Junsun Hwang, Dara Bayat, Thomas Overstolz, Amit Dolev, Sarah Heub, Gilles Weder, Michel Despont and Mahmut Selman Sakar

Supplementary Figures

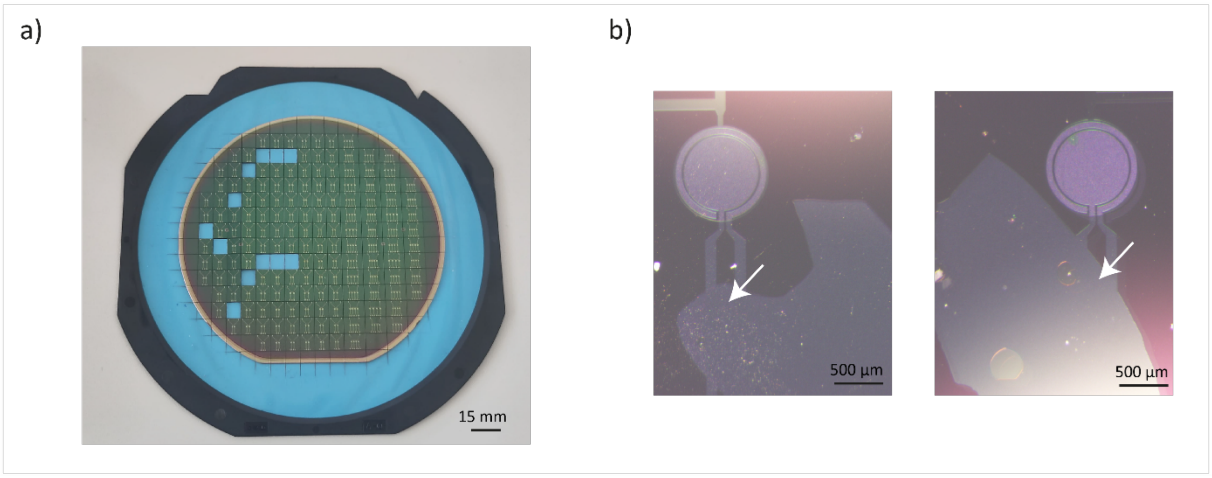

Fig. S1. PMUT array fabrication. a) 6-inch wafer containing 115 PMUT arrays of different designs. Some arrays were already retrieved for characterization. b) Photographs showing typical defects (white arrows) on the PMUTs. The electrodes tracks were damaged during their patterning or the deposition of the electrical insulation layer.

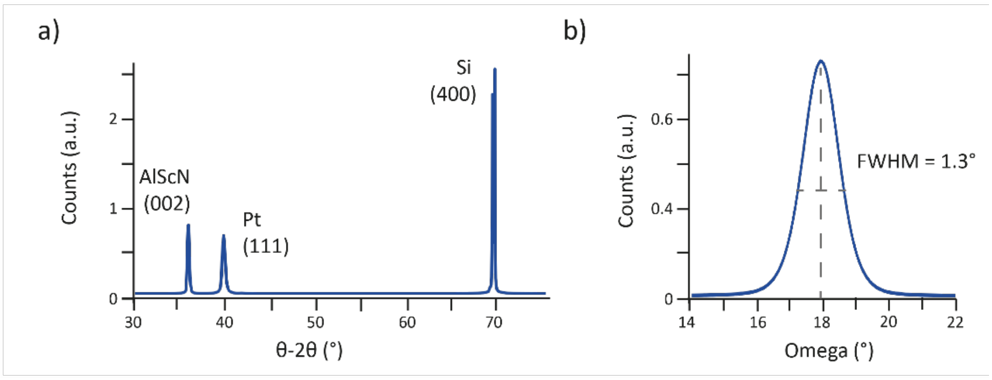

Fig. S2. Characterization of the AlScN piezoelectric thin film. a) Material composition of the deposited AlScN piezoelectric layer measured by X-ray crystallography analysis. b) Rocking curve corresponding to the AlScN peak shown in (a) reveals the crystalline quality of the film.

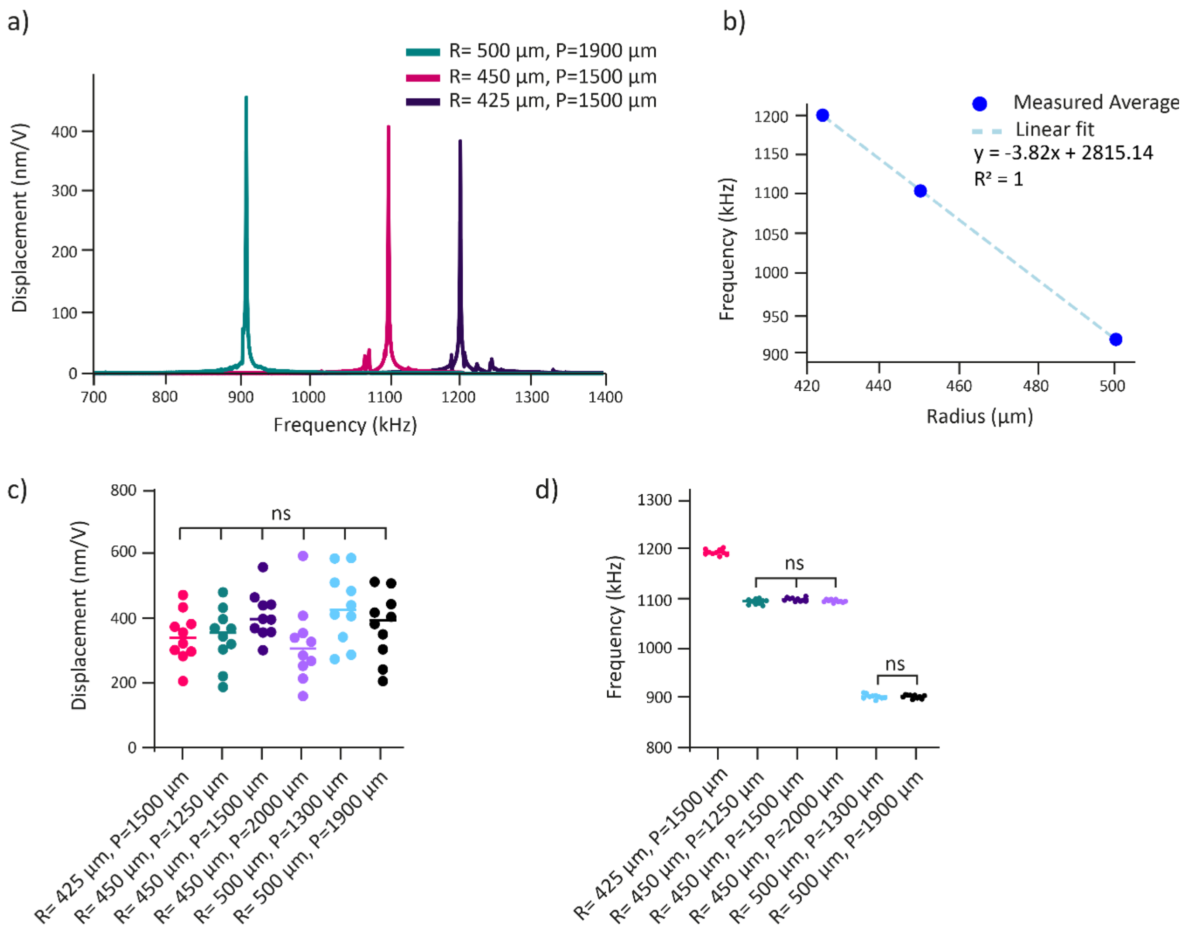

Fig. S3. Vibrational characterization of PMUTs in air with varied PMUT radius (R) and pitch (P). a) Frequency response of PMUTs with different radii. b) Resonance frequency as a function of PMUT radius. c) Maximal displacement amplitude at the fundamental resonance frequency for various array designs with differing radii and pitches. No statistically significant difference was observed across the designs. d) Resonance frequency of PMUTs for different array designs. Arrays with identical PMUT radii but varying pitch show no statistically significant variation in resonance frequency.

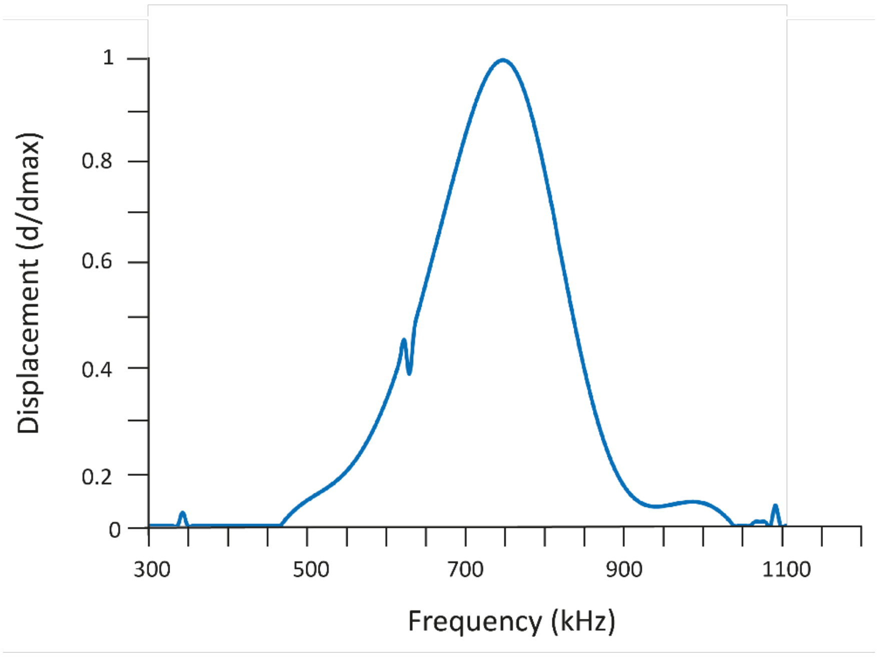

Fig. S4. Simulated fundamental resonance frequency of a PMUT with a radius of 450 µm and thickness of 80 µm submerged in water.

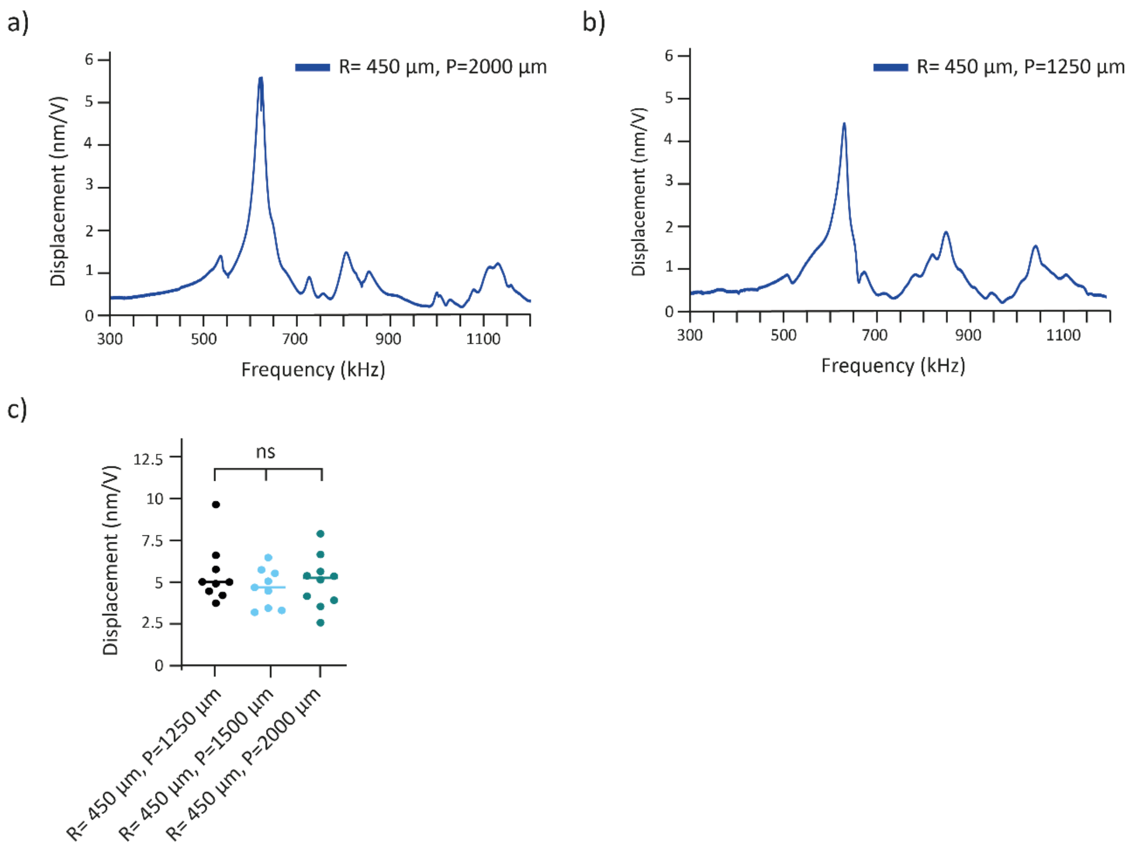

Fig. S5. Vibrational characterization of PMUTs in water for different array designs with a unique PMUT radius (R: 450 µm) but with varied pitch (P). a-b) Frequency response of a PMUT with different pitches: (a) pitch = 2000 µm and (b) pitch = 1250 µm. c) Maximum displacement at the fundamental resonance frequency for different pitches. There is no statistical difference between all the designs.

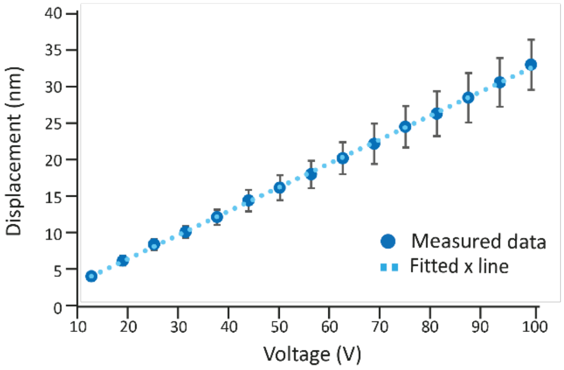

Fig. S6. Linear response of PMUT diaphragm displacement in H2O as a function of the voltage. The measurements were done at frequencies between 630 kHz and 625 kHz.

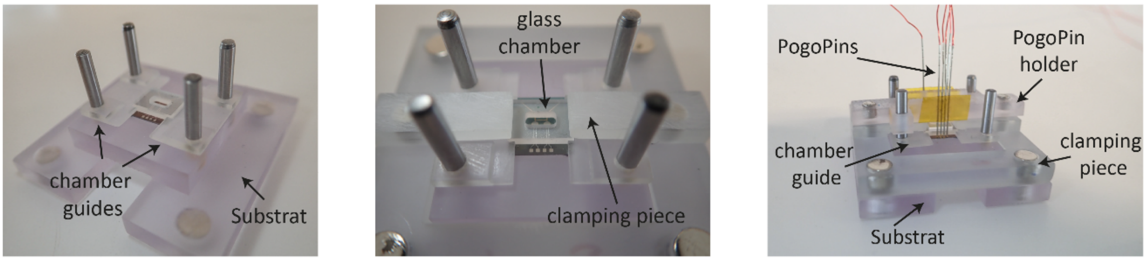

Fig. S7. Components of the custom clamping setup. Photographs showing the individual components of the custom-designed clamping system used for PMUT array operation. The setup consists of five parts: one substrate, two chamber guides, one clamping piece, and one PogoPin holder. Each component is designed to ensure precise alignment, secure sealing, and reliable electrical interfacing during experimental measurements.

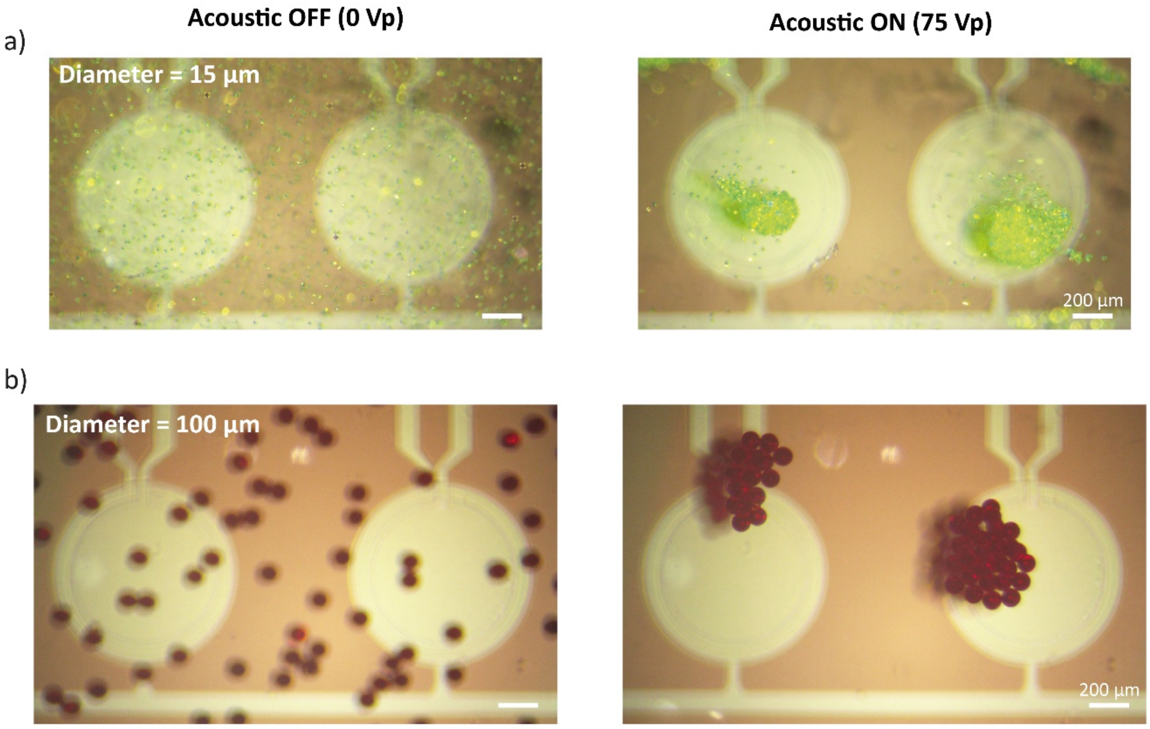

Fig. S8. Acoustic trapping of PS particles with diameter of (a) 15 µm and (b) 100 µm on top of the PMUTs using a driving signal of 620 kHz and 75 Vp.

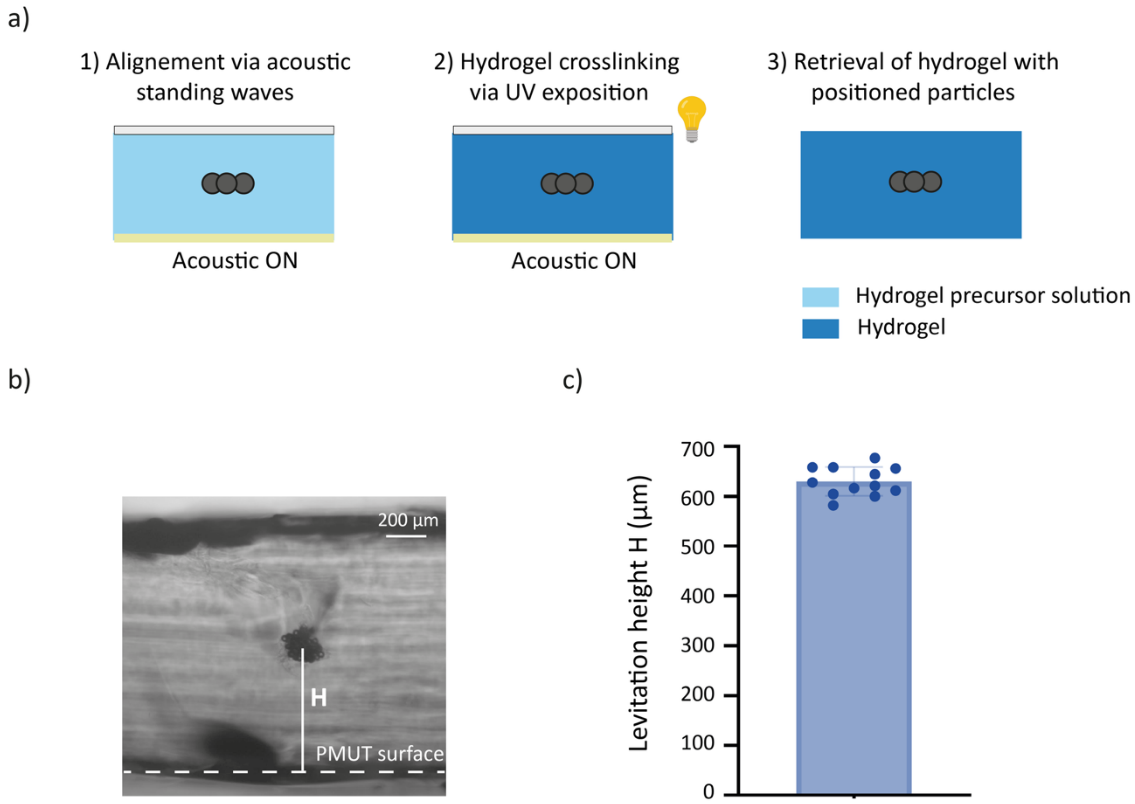

Fig. S9. Levitation of particles inside a hydrogel. a) Schematic illustrating the positioning of particles in a hydrogel precursor using acoustic waves. b) Cross-section of a hydrogel after crosslinking, showing one levitated PS particle aggregate. H denotes the levitation height. c) Mean and standard deviation of H measured across 3 devices and 12 samples.

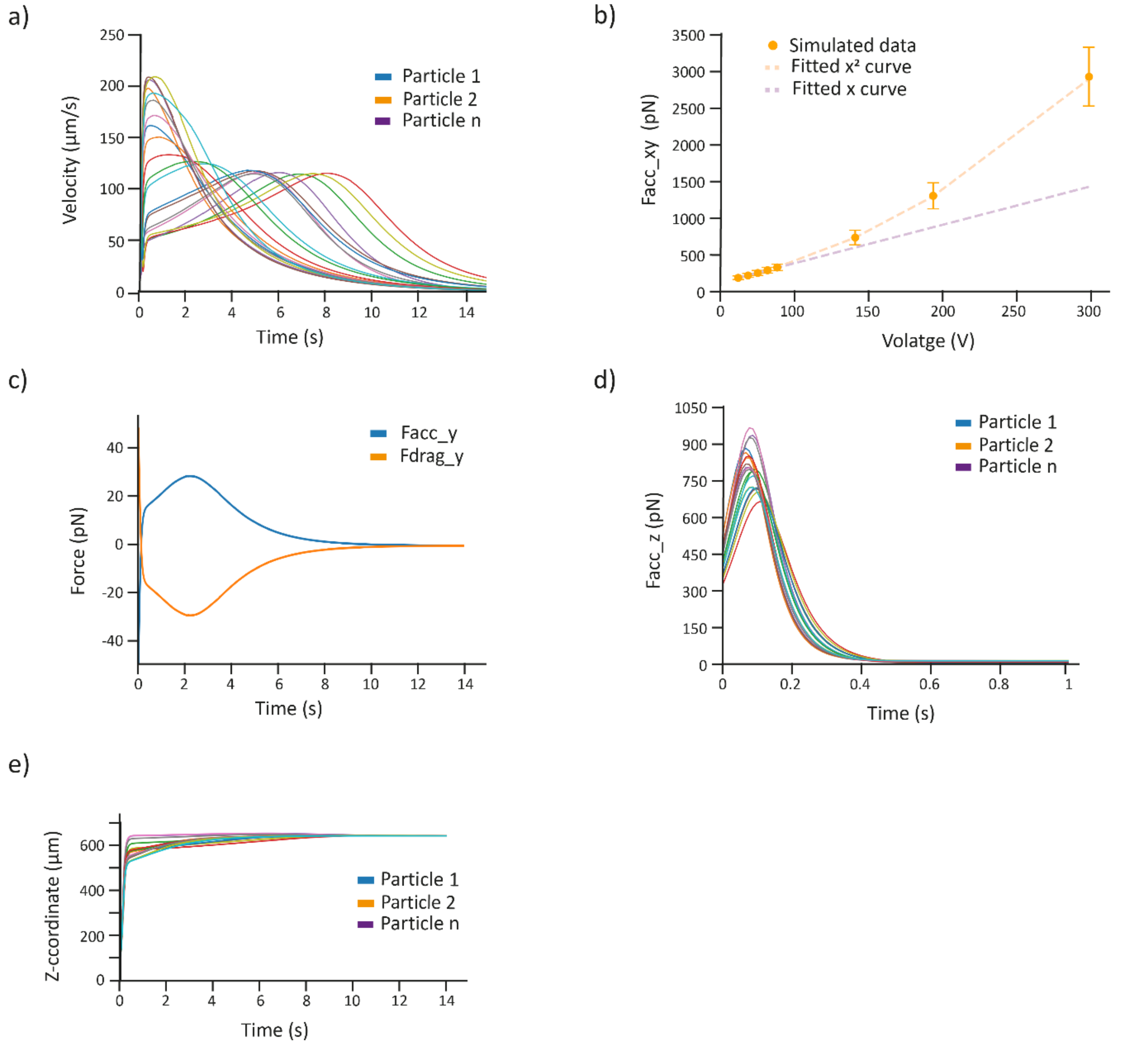

Fig. S10. Simulated variables describing particle trajectories during acoustic manipulation. a) In-plane velocity as a function of time for 20 particles at 75 V_p excitation. b) Maximum in-plane velocity of particles as a function of applied voltage. Each data point represents the average maximum velocity across 20 particles. The observed trend confirms a quadratic dependence on the excitation voltage. c) Comparison between the in-plane acoustic radiation force and hydrodynamic drag force for a single particle at 75 V_p, highlighting their equivalence. d) Vertical acoustic radiation force for 20 particles during acoustic trapping. e) Time evolution of the z-position for 20 particles, showing vertical localization of the acoustic trap at 75 V_p.

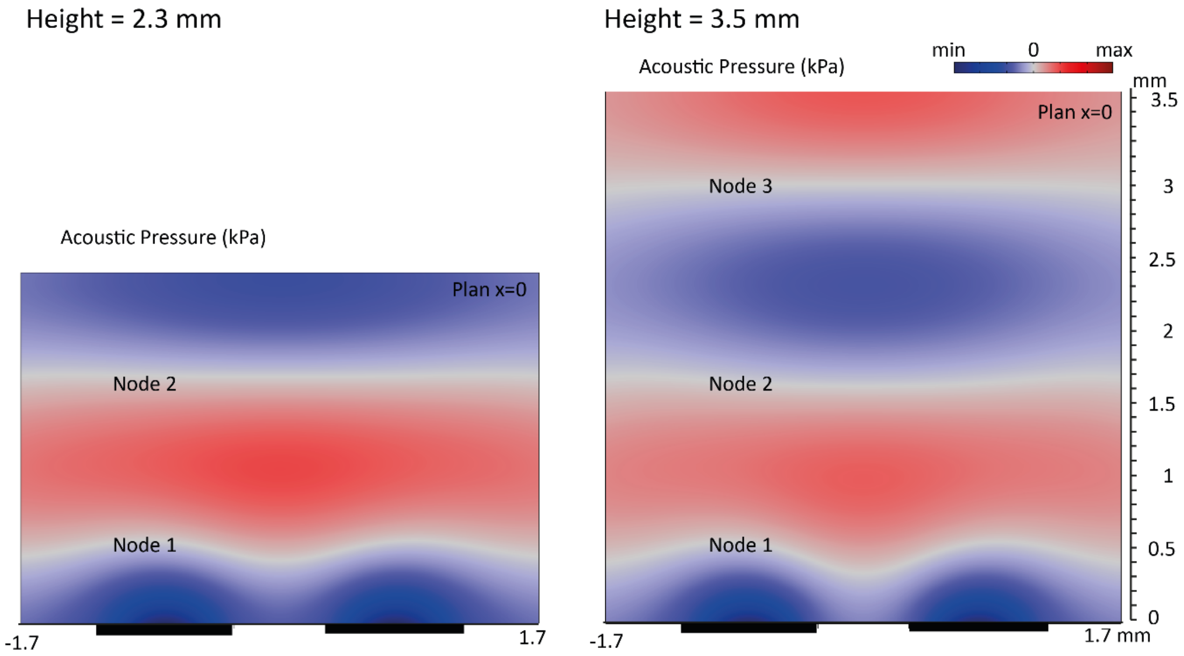

Fig. S11. Effect of chamber height on the generation of multiple acoustic nodes. (left) Simulation showing the formation of two acoustic pressure nodes at 620 kHz when the chamber height is increased to 2.3 mm. (right) Simulation showing the formation of three acoustic pressure nodes at 620 kHz when the chamber height is increased to 3.5 mm.

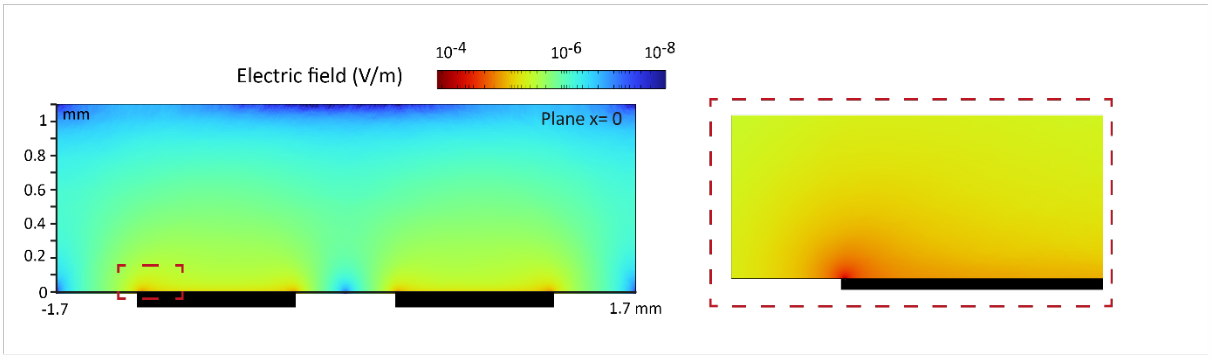

Fig. S12. Simulated electric field for an input voltage of 75 V_p for the vertical cross-section plane x = 0. The colour scale is logarithmic.

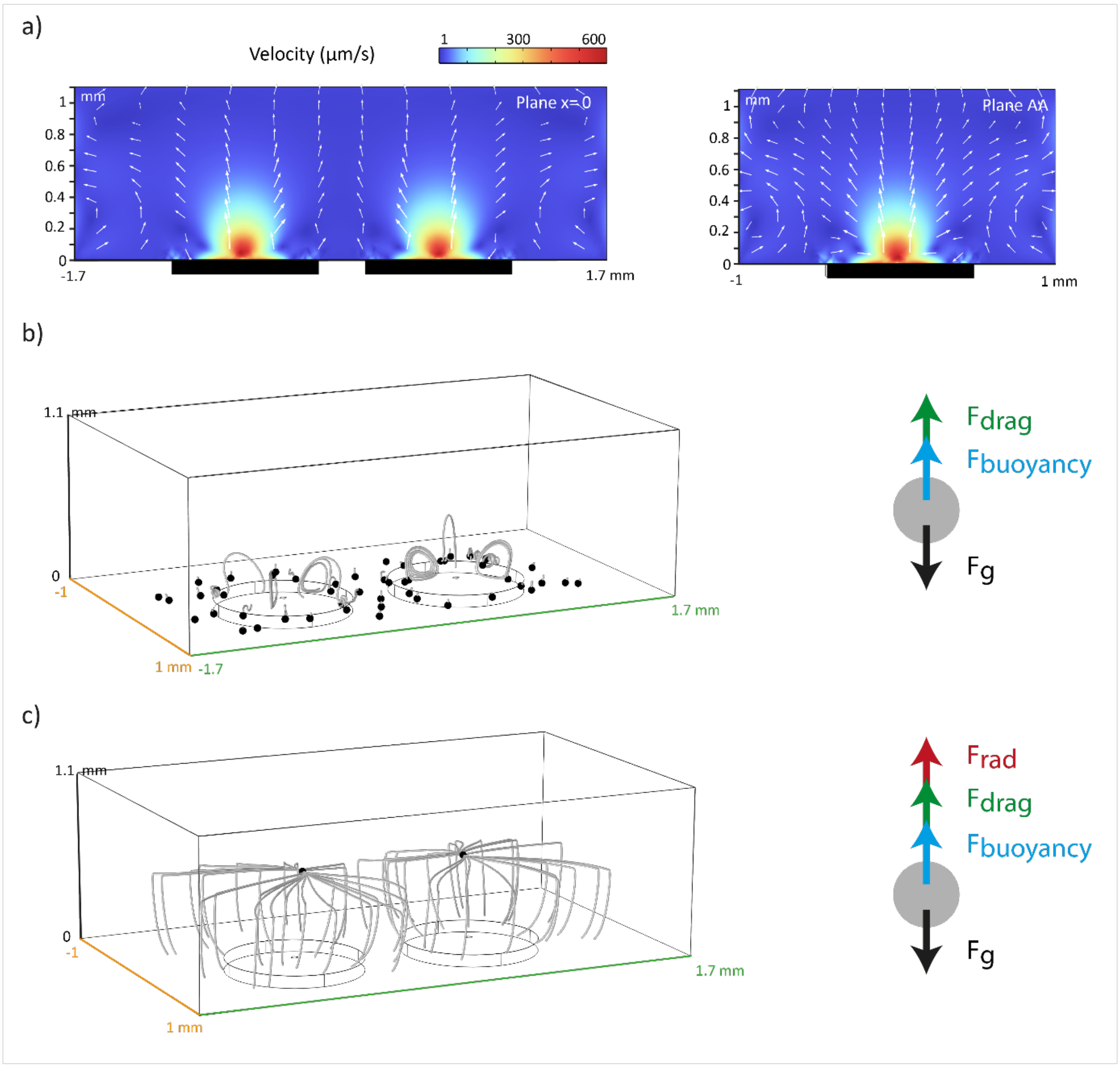

Fig. S13. a) Simulated acoustic streaming at an input voltage of 75 V_p, shown in the vertical cross-sectional planes x = 0 and AA (as defined in Figure 5b). White arrows indicate the direction of the velocity vectors. b) Simulated trajectories of PS particles (30 µm diameter) initially in a sedimented state, considering only the drag force from acoustic streaming and gravity. The results indicate that acoustic streaming alone is insufficient to lift or manipulate the particles. c) Simulated trajectories of PS particles (30 µm diameter) under the combined influence of acoustic streaming-induced drag, acoustic radiation force, and gravity. The trajectories closely resemble those observed without streaming (see Figure 4b and Supplementary Video 2), suggesting that acoustic streaming has negligible influence on particle motion in this configuration.

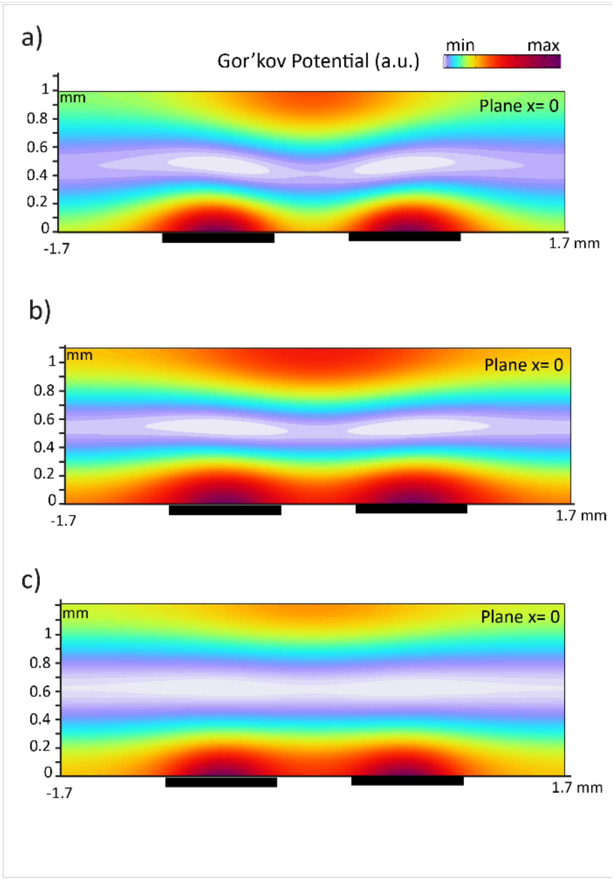

Fig. S14. Simulated Gor'kov potential for different chamber height. a-c) Gor'kov potential for the vertical cross-section plane x = 0 for (a) h = 1000 µm, (b) h = 1100 µm and (c) h = 1200 µm. Dark rectangles indicate PMUT locations.

Supplementary Table

Table S1 Expression of functions and parameters used in the FE simulation using COMSOL MULTIPHYSICS.

Name	Expression	Unit	Description
**U_gor**	(f1Gor-f2Gor)	Pa = J/m³	Gor'kov potential energy density
**f1Gor**	(f1*.5*realdot(acpr.p_t,acpr.p_t))/(2*acpr.rho*c0^2)	Pa= J/m³	
**f2Gor**	0.75*f2*acpr.rho*.5*(realdot(acpr.vx,acpr.vx)+realdot(acpr.vy,acpr.vy) + realdot(acpr.vz,acpr.vz))	Pa= J/m³	
**f1**	1-(k_p/k_m)		
**f2**	2*((rho_p-acpr.rho)/(2*rho_p+acpr.rho))		
**rho_p**	1100	kg/m³	Density PS particles[1]
**c0**	1480	m/s	Speed of sound in water[2]
**k_p**	2.47E-10	Pa	Compressibility PS particles[1]
**k_m**	4.56E-10	Pa	Compressibility water[2]
**cL_p**	2350	m/s	Longitudinal (pressure) wave speed PS particles[1]
**cT_p**	1120	m/s	Transverse (shear) wave speed PS particles[1]

References

1. Settnes, M. & Bruus, H. Forces acting on a small particle in an acoustical field in a viscous fluid. *Phys. Rev. E* **85**, 016327 (2012).
2. Modeling an Acoustic Trap: Thermoacoustic Streaming and Particle Tracing. *COMSOL* https://www.comsol.com/blogs/modeling-an-acoustic-trap-thermoacoustic-streaming-and-particle-tracing.

Supplementary Videos

Video S1: Acoustic trapping of 30 µm-diameter PS particles in PMUT-based platform.

Video S2: Acoustic trapping of 100 µm-diameter PS particles in PMUT-based platform.

Video S3: Simulated trajectories of PS particles in PMUT-based platform.

Video S4: In-plane trajectories of PS particles.

Video S5: Acoustic trapping of PS particles in two traps (h = 1000 µm).

Video S6: In-flow acoustic trapping of PS particles.

Video S7: Limits of in-flow acoustic trapping.

Video S8: Bidirectional translation of PS particles between adjacent PMUTs.

Video S9: Translation and merging of PS particle aggregates between adjacent PMUTs.

Video S10: Splitting of PS particle aggregate between adjacent PMUTs.
